# Experience of Saving Limbs With Free Fibula Osteocutaneous Flaps

**DOI:** 10.7759/cureus.16600

**Published:** 2021-07-24

**Authors:** Fahad H Khan, Obaid Rahman

**Affiliations:** 1 Plastic and Reconstructive Surgery, Liaquat National Hospital and Medical College, Karachi, PAK

**Keywords:** hand and feet reconstruction, free fibula flap, osteo-cutaneous flap, trauma, oncological reconstruction

## Abstract

Background

Complex wounds of hands and feet have always been a challenge for reconstructive surgeon. We aim to share our experience of reconstruction of such defects using free fibula osteocutaneous flaps.

Materials and methods

This is a retrospective study over a period of six years which was conducted at a tertiary care teaching hospital in Karachi. All patients, who were included and had reconstruction with this flap, agreed to participate in this study. Hospital records were retrieved for patient’s demographic details, mode of injury, size of the defect, number of bone loss in the defect, dimensions of flap, size and number of bony segments in each flap. Outcomes were recorded in terms of flap survival and secondary procedures, with post-operative radiographs.

Results

In 14 patients, 25 (80.5%) metacarpals and 6 (19.5%) metatarsals defects were reconstructed. K-wires were used for bony stabilization and were removed at 8 weeks post-operatively. Only two flaps were re-explored due to venous congestion. Minor wound dehiscence was noted in two flaps which were managed conservatively.

Conclusion

Proper planning and meticulous flap dissection and inset using free fibula flap can save many hand and foot from amputations

## Introduction

Composite defect of hand and foot, secondary to trauma or after removal of tumor usually includes metacarpals or metatarsals with tendon nerves and vessels and skin, is a reconstructive dilemma. General practice is to first cover the soft-tissue defect then grafting of bone, tendon and nerves. Reconstruction of these composite defects with osteocutaneous free flap is also an option followed by reconstruction of tendons and nerves in later stages.

Unlike other flap options of osteocutaneous free flap, free fibula flap can be harvested by techniques described by Hidalgo [1] with very reliable one or more skin pedicles, well-vascularized long thin bone which can easily be harvested in segments, a relatively long and good-sized vascular pedicle, relatively short learning curve and minimum donor site morbidity. Due to these benefits we use free fibula as our first option in osteocutaneous defects and through this study, we aim to share our experience in hand and foot osteocutaneous reconstruction with free fibula flap.

## Materials and methods

This is a retrospective study over a period of six years, from January 2012 to December 2017, conducted at the Department of Plastic and Reconstructive Surgery at a tertiary care hospital in Karachi. This hospital is the biggest private sector teaching hospital in the city, with all multidisciplinary facilities. It has played a key role in providing basic and advanced health care to the patients from all over Pakistan and from neighboring countries, especially from war-afflicted regions of Afghanistan. As a teaching institute, we inform and take written consent from our patients for the possible use of data for research purpose, while keeping their identity anonymous. The study was approved by the institute’s review board.

We retrieve our data of free vascularized fibula used in metacarpal and metatarsal reconstruction and assessed it for demographic profile, mode of injury, size of defect, number of bone loss in defects, dimensions of flap, length and number of bone segments in each flap, survival and secondary procedures performed. Outcomes were assessed by the viability of bone on X-ray images after 3 months.

Flap dissection

**Figure 1:**
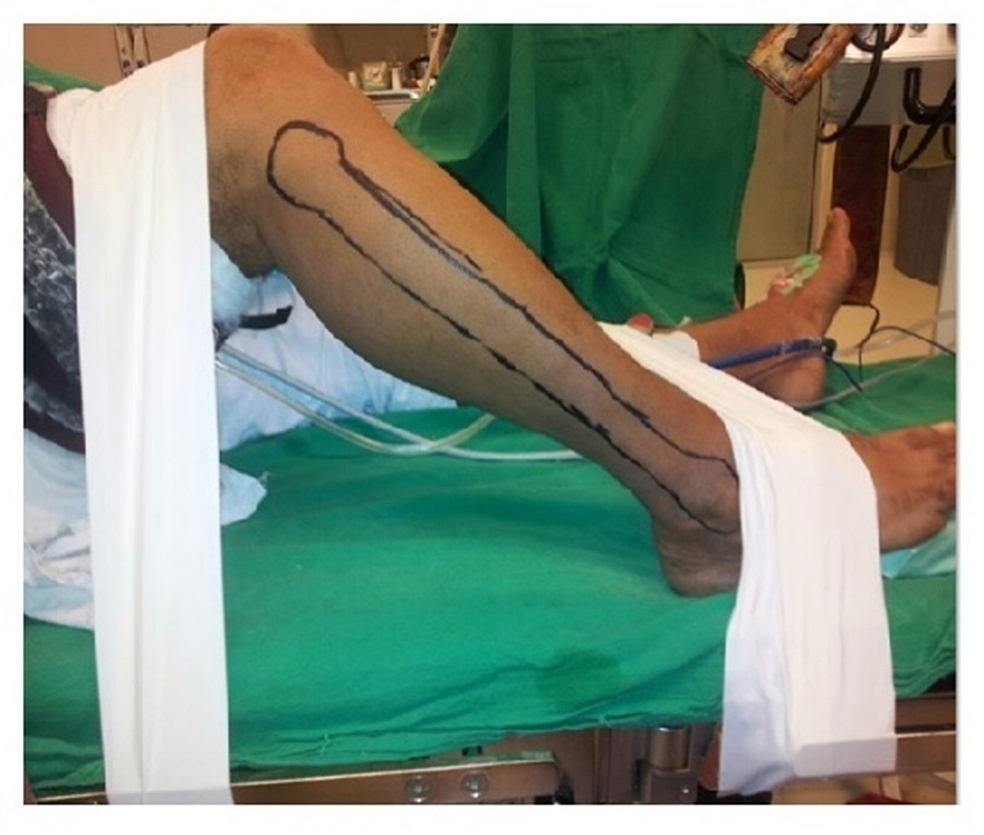
Operative position of the leg using tapes and soft paddings for easy harvesting of vascularized fibular flap.

After recreating the final defect in traumatic patients or achieving frozen section clearance in oncological patients, we measured final dimensions of skin and bony defect using a standard centimeter scale. Flap was carefully planned on right leg, which was surgeon’s preference in all cases. Patient remained in supine position and donor leg was fixed to table by adhesive tapes to facilitate the harvest (Figure 1). Fibula was mapped and skin island was marked, centered on perforator using 8 Mhz (Huntleigh - D900) handheld doppler probe. In only one case, two skin islands were required to cover both volar and dorsal surface of hand.

Flap was harvested under tourniquet control and with 4x loupe magnification. Incision was given on the anterior aspect of skin pedicle and deepened down to the muscles of anterior compartment. Skin island was elevated off the tibialis anterior, the extensor digitorum longus muscles of the anterior compartment and then the peroneus longus and brevis muscles of the lateral aspect of the fibula preserving the superficial peroneal nerve. On confirmation of adequate septocutaneous perforators the posterior skin margin was incised. The sural nerve and lesser saphenous vein were preserved. The skin island was elevated off the lateral gastrocnemius and soleus muscles to the posterior edge of the fibula. After dissecting peroneus longus and brevis off the fibula, osteotomies were performed leaving 6 centimeters of fibula at ankle joint. Next, the anterior compartment muscles, including the tibialis anterior, extensor digitorum longus and extensor hallucis longus were identified and dissected from the fibula and retracted. The interosseous membrane was divided along its length. Distal peroneal vessels were identified and ligated. Tibialis posterior muscle was dissected of the bone preserving the underlying peroneal vessels. A cuff of flexor hallucis was left attached to fibula, rest of gastrocnemius and soleus were dissected from the fibula. Pedicle was dissected upto tibioperoneal trunk to gain maximum length.

Once vascularity of both bone and skin were checked by deflating the tourniquet custom osteotomies were performed and flap was transferred to its recipient site. Once bony fixation was achieved with K (Krishner) wires, tagging sutures were applied on skin and anastomosis performed under microscope. Flap inset was done leaving penrose drains in situ. Loose soft dressing was applied and a short-term splint was applied. Monitoring and management of free flap were done according to our departmental protocol with a very low threshold for turn back to the operating room.

## Results

We treated 14 patients using free fibula for the reconstruction of metacarpals and metatarsals, in which male to female ratio was 6:1. The mean age was 30.9 ± 8.3 years (range, 17-43). Our 11 (78.5 %) patients had defects due to trauma while 3 (21.5%) patients had defects secondary to tumor resection.

Twenty-five (80.5%) metacarpals and 6 (19.5%) metatarsals defects were reconstructed. A total of 31 bone defects were reconstructed using 28 bony segments. Mean length of bony segments were 3.1 ± 1.2 centimeters (cm). In one patient, we reconstructed using two skin pedicles of 5 x 7 cm and 4 x 3 cm, rest of our 13 patients required one skin pedicle with a minimum dimension of 2 x 5 cm and maximum dimensions of 9 x 16 cm.

Only two flaps were reexplored due to venous congestion, out of them one flap with two skin pedicles had a loss of skin pedicle and we covered it with a staged groin fascio-cutaneous flap. The other flap survived after the evacuation of hematoma. Minor wound dehiscence was noted in two flaps which were managed conservatively.

Outcome

Thirteen (92.8 %) flaps survived with a mean follow-up of 14 months (range: 3-22 months). K-wires were removed in all cases at eight weeks and all of the reconstructed hands and feet were allowed to do daily life activity after three months. Most of our patients underwent secondary procedures including extensor tendon reconstruction with tendon grafts (7/13), arthrolysis (6/13), two-staged flexor tendon reconstruction (2/13), nerve grafts (2/13) and resuturing of wound (1/13). All patients achieved reasonably good functional recovery.

## Discussion

Composite reconstruction is a challenging task as it requires proper planning and execution. Many authors had used free fibula flap as their first choice for the reconstruction for mandible [2,3], maxilla [4,5], clavicle [6], upper limb and lower limb [7,8], epiphysial transfer [9], hand [10] and foot [11-13]. One recent study by Taylor et al. who also reported the first free vascularized fibula for a lower limb trauma, discuss many uses of vascularized fibula with his 40 years of experience [11,14]. There are other options of vascularized osteo-cutaneous flap include scapula, rib, radius as summarized by Houdek et al. [12] and medial condyle of femur and metatarsal [13,15,16]. Problems associated with other options are short pedicle length, unreliable and bulky skin pedicel, limited bone, limited segments of bones and donor site deformity.

 At our institute, free fibula is a workhorse flap for the reconstruction of mandible and other osteo-cutaneous defects. For the smaller defects like metacarpals and metatarsals, we use to do bone grafting after soft tissue coverage but we consider free fibula in the setting of tumor excision where there is a possibility of postoperative radiation as vascularized bone can tolerate radiation better with good bone healing [17]. We also consider single-stage reconstruction in patients who do not want groin flap or multiple procedures for the reconstruction of complicated hand trauma. We lack the facilities of three-dimensional virtual planning of fibular flap for metacarpal as reported by Shen et al [18].

**Figure 2:**
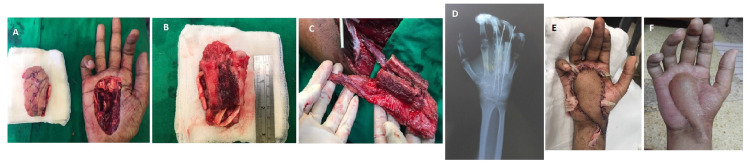
Defect after tumor excision (A), resulting in loss of metacarpals, tendons, neurovascular bundles and skin (B). Vascularized osteocutaneous flaps with two bony segments, adequate skin and long vascular pedicle (C). Bones were stabilized with K-wires (D). Post-operative result after 24 hours (E) and six months post adjuvant therapy (F).

One of our patients had a defect in his dominant right hand after tumor excision (Figure 2). Tumor clearance was confirmed by intraoperative frozen section, reconstruction was done using double-barrel free vascularized fibula to reconstruct 3rd and 4th metacarpal and skin coverage. Bones were fixed using K-wires and anastomosis done with radial artery and cephalic vein. This patient had post-operative radiation and had good vascularized coverage and healed bone at six months post operatively.

**Figure 3:**
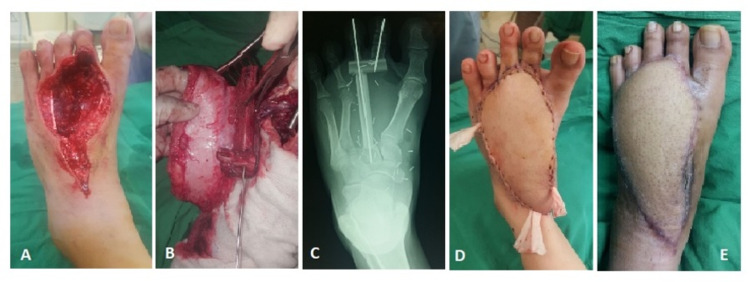
Defect on foot after tumor excision (A). Osteotomies and realignment of segments in L-shape to provide pillar support for two toes (B and C). Post-operative result after 24 hours (D) and six months post adjuvant therapy (E).

Another patient had a similar defect in her foot (Figure 3). After tumor clearance reconstruction with free fibula flap was done using an L-shaped bony structure, and adequate skin flap. Bony fixation was achieved using K-wires and anastomosis was done with dorsalis pedis artery and saphenous vein.

In our data, all patients had good bony union as well as stable skin coverage except in one patient where skin pedicles were debrided and skin cover was provided with groin flap. After multiple secondary procedures, all hands and feet were functional. Despite many benefits of free fibular flaps we encounter few donor site morbidities as well. Two of our patients had less than 10% graft loss at donor area, three patients had weakness of flexor hallucis longus (MRC score: 4/5) and one patient had a painful neuroma of sural nerve [19]. None of our patients had gait-related issues as already reported by Ferrari et al. [20].

## Conclusions

From our experience, free fibula flap is a good option for the reconstruction of hand and foot. Proper planning and meticulous flap dissection and inset can save many hand and foot from amputations.
